# Fluid and electrolyte imbalance related to intracranial abnormality

**DOI:** 10.1186/1687-9856-2013-S1-P201

**Published:** 2013-10-03

**Authors:** Frida Soesanti, Bambang Tridjaja, Jose RL Batubara, Aman Pulungan

**Affiliations:** 1 Pediatric Endocrinology Division, Faculty of Medicine, Universitas Indonesia, Ciptomangunkusumo Hospital, Jakarta, Indonesia

## Background

Maintenance of the tonicity of extracellular fluids is crucial for proper cell function. In children and adults, normal blood tonicity is maintained by a coordinated interaction among the thirst, vasopressin, and renal systems. Dysfunction in any of these systems can result in abnormal regulation of blood osmolality, which if not properly recognized and treated, may cause life-threatening dysfunction in neuronal and other cellular activities. The aim of this study is to increased awareness on the possibility of fluid and electrolyte imbalance in patients with intracranial abnormality.

## Case series

There were five cases consulted to endocrinology division due to polyuriaduring February-June 2012. All of them had intracranial abnormality; Langerhans cell hystiocytosis (LCH),cerebellimeduloblastoma, retinoblastoma with intracranial metastases, pituitary adenoma post extirpation, and intracranial abscess. During admission these patients develop polyuria, ranging from 6 to 16.6 mL/kg/hr. Etiology of polyuria were central diabetes insipidus in three patients (LCH, Meduloblastoma, pituitary adenoma patients), and CSW in two patients, the rate of urine were significantly higher in CDI than CSW cases. In CDI, the urine gravity was <1.005, the serum sodium was 138-142 mEq/L with urine sodium of 8-36 mEq/L, and the serum osmolality significantly higher than urine osmolality. None of our patient needed water deprivation test to confirm the diagnosis. Those with CDI were treated with DDAVP nasal spray. None of our patient had water intoxication due to DDAVP; instead the dose should be increased to 3x20 mcg in one patient. In the CSW patients, serum sodium level was quite low, reached 110 mEq/L, manifest as seizure, and unresponsive to rapid sodium corrections. In these patients, the urine gravity was normal and urine sodium excretion was>140 mEq/L. None of these patients had specific signs of CSW. We increased sodium intake up to 10 mEq/kg/day and maintained the fluid balance to prevent dehydration. One patient received fludrocortisone for one week. Four of 5 patients outcomes were good, but one patient died due to multiorgan failure associated with main disease.

## Conclusion

Polyuria and electrolyte imbalance are not rarely occur during the course of other diseases, especially those with underlying intracranial pathology. Early identification and treatment of these condition will reduce morbidity and mortality associated with fluid and electrolyte imbalance and increase overall treatment outcome.

